# Detection of SHV β-lactamases in Gram-negative bacilli using fluorescein-labeled antibodies

**DOI:** 10.1186/1471-2180-9-46

**Published:** 2009-03-02

**Authors:** Andrea M Hujer, Karen S Keslar, Nicole J Dietenberger, Christopher R Bethel, Andrea Endimiani, Robert A Bonomo

**Affiliations:** 1 Research Service, Louis Stokes Cleveland Department of Veterans Affairs Medical Center, Cleveland, OH, USA; 2 Department of Medicine, Section of Infectious Diseases, Case School of Medicine, Cleveland, OH, USA; 3 Department of Pharmacology, Case School of Medicine, Cleveland, OH, USA; 4 Department of Molecular Biology, Case School of Medicine, Cleveland, OH, USA; 5 Department of Microbiology, Case School of Medicine, Cleveland, OH, USA

## Abstract

**Background:**

β-lactam resistance in Gram-negative bacteria is a significant clinical problem in the community, long-term care facilities, and hospitals. In these organisms, β-lactam resistance most commonly results from the production of β-lactamases. In Gram-negative bacilli, TEM-, SHV-, and CTX-M-type β-lactamases predominate. Therefore, new and accurate detection methods for these β-lactamase producing isolates are needed.

**Results:**

*E. coli *DH10B cells producing SHV-1 β-lactamase and a clinical isolate of *K. pneumoniae *producing SHV-5 β-lactamase were rendered membrane permeable, fixed and adhered to poly-L-lysine coated slides, and stained with purified polyclonal anti-SHV antibodies that were fluorescein labeled. *E. coli *DH10B cells without a *bla*_SHV _gene were used as a negative control. The procedure generated a fluorescence signal from those slides containing cells expressing SHV β-lactamase that was sufficient for direct imaging.

**Conclusion:**

We developed a rapid and accurate method of visualizing the SHV family of enzymes in clinical samples containing Gram-negative bacilli using a fluorescein-labeled polyclonal antibody.

## Background

Resistance to β-lactam antibiotics in Gram-negative bacteria is a significant clinical problem in the community, long-term care, and hospital settings [1-3]. In the common Gram-negative bacteria that are responsible for most clinical infections, β-lactam resistance results from production of penicillinases (predominantly the β-lactamases designated TEM-1 and SHV-1), cephalosporinases (e.g., extended-spectrum β-lactamases, ESBL, of TEM-, SHV- and CTX-M-types), and the chromosomally or plasmid encoded AmpC enzymes [1]. Hence, an aggressive search for novel therapeutic agents and rapid, accurate detection methods is necessary.

Polymerase chain reaction (PCR) based techniques (such as multiplex PCR, real time PCR, DNA microarrays) and DNA-DNA hybridization have been used with success to detect *bla *genes in Gram-negative bacilli [4-10]. Most recently, fluorescence *in situ *hybridization (FISH) using rRNA oligonucleotides has also been employed to detect β-lactamase genes [11,12]. Unfortunately, not all clinical microbiology laboratories can perform the above molecular techniques. Even if available, these methodologies are not routinely used to study clinical samples because they are expensive and time consuming. We would also emphasize that a PCR amplification product indicates the presence of the gene only and does not always indicate protein production.

In a previous study, our laboratory raised and characterized polyclonal antibodies against the SHV-1 β-lactamase [13,14]. Immunogenic epitope mapping of the SHV β-lactamase was reported. The polyclonal antibodies detected as little as 1 ng of β-lactamase by immunoblotting and pg quantities by enzyme-linked immunosorbent assay (ELISA). Notably, cross reaction with other class A β-lactamases (i.e., TEM- and CMY-2-like enzymes) was not observed [13,14].

In this report, we extend our investigations and describe a method using fluorescein-labeled polyclonal antibodies (FLABs) to visualize the SHV-type β-lactamases expressed in a laboratory strain of *Escherichia coli *and in a clinical isolate of *Klebsiella pneumoniae*. With this technique, we have developed a new method by which we could rapidly detect SHV-type β-lactamases in clinical samples using FLABs and fluorescence microscopy.

## Methods

The SHV-1 β-lactamase gene was sub-cloned into the pBC SK(-) vector (Stratagene, LaJolla, CA) from a clinical strain of *K. pneumoniae *(15571), and transformed into *E. coli *DH10B cells (Invitrogen, Carlsbad, CA) [15]. The *K. pneumoniae *clinical isolate possessed the SHV-5 ESBL and was obtained from a previous study [16]. *E. coli *DH10B without the *bla*_SHV-1 _gene served as a negative control.

The procedures used to isolate, express and purify the SHV-1 β-lactamase and to produce the anti-SHV β-lactamase antibodies have been previously detailed [13]. Purified anti-SHV antibodies were fluorescein-labeled with the EZ-Label™ fluorescent labeling kit (Pierce, Rockford, IL), according to the instructions of the manufacturer. In brief, 1 mg of polyclonal anti-SHV antibodies in 1 ml phosphate buffered saline (PBS, 2 mM monobasic sodium phosphate, 8 mM dibasic sodium phosphate, 154 mM sodium chloride, pH 7.4) was mixed with 7.6 μl of a 10 mg/ml solution of NHS-fluorescein in N, N-dimethylformamide for 1 hr at room temperature. A desalting column was then used to separate unbound fluorescein from labeled antibodies. Labeled antibodies exiting the column were monitored by measuring the absorbance of the samples at 280 nm. Then, the labeled antibodies were filter-sterilized, protein concentration determined, and stored at 4°C.

*E. coli *DH10B with and without the *bla*_SHV-1 _gene in the pBC SK(-) phagemid vector and the clinical isolate of *K. pneumoniae *possessing the SHV-5 β-lactamase were prepared for staining and visualization by fluorescence microscopy on a Zeiss Axiovert 200 inverted scope. Stationary phase cells were grown to 37°C in Luria Bertani broth supplemented with either 20 μg/ml of chloramphenicol (Sigma, St. Louis, MO) or 50 μg/ml ampicillin (Sigma), for *E. coli *DH10B harboring the *bla*_SHV-1 _gene or the clinical isolate of *K. pneumoniae*, respectively. Antibiotics were not used in the case of *E. coli *DH10B cells alone. Overnight cultures were diluted to an OD_600 _nm of 0.5 and 500 μl of cells were spun down and re-suspended in 500 μl of 50 mM Tris HCl, pH 7.4. Lysozyme was added to a final concentration of 1 mg/ml for 5 min, followed by addition of 1 mM EDTA for 5 min. Cells were then pelleted (5000 g × 5 min), washed twice with PBS, and re-suspended in 500 μl of PBS. Cells were fixed with the addition of 100 μl of fixation buffer containing 200 mM dibasic sodium phosphate, 0.3% glutaraldehyde, and 2% formaldehyde (Sigma). Cells were incubated for 10 min at room temperature and then for 45 min on ice, washed once with PBS, and re-suspended in 250 μl PBS. Ten μl of this suspension was placed on a poly-L-lysine coated slide (Sigma), and after 1 min the liquid was aspirated off. The adhered cells were gently washed once with 50 μl of PBS and then the specimen was allowed to dry completely.

The fixation procedure was followed by re-hydration and staining. Each cell-adhered area was re-hydrated by adding 100 μl of PBS for 4 min followed by aspiration. Each area was then blocked with 2% bovine serum albumin (BSA), in PBS for 15 min at room temperature in a humidity chamber, followed by aspiration. A 5 μg/ml solution of FLABs in 2% BSA in PBS was then added and allowed to incubate for 2 hrs in the humidity chamber. The cells were then washed 10 times with PBS, the excess liquid was aspirated off, and 2–3 drops of Gelmount was added followed by the addition of a coverslip. The procedure aimed for a fluorescence signal sufficient for imaging directly. The Zeiss Axiovert 200 inverted scope was equipped with an Axiocam digital microscope camera to capture immunofluorescence images.

## Results and discussion

**Figure 1:**
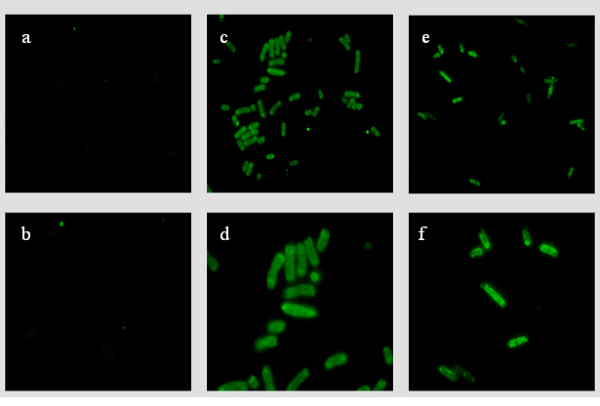
**a and b: *E. coli *DH10B cells devoid of SHV β-lactamase stained with anti-SHV FLABs**. c and d: detection of periplasmic SHV β-lactamase in a *K. pneumoniae *clinical isolate possessing the SHV-5 β-lactamase. e and f: visualization of SHV-1 β-lactamase in a laboratory strain of *E. coli *expressing SHV-1. Microscopic magnification is 1000×. Figure 1b, 1d, and 1f are enlarged images.

Figure 1 demonstrates the immunofluorescence images obtained using the fluorescence microscope at 1000 × total magnification. Figure 1a and 1b show *E. coli *DH10B cells devoid of SHV β-lactamase stained with anti-SHV FLABs. In Figure 1c and 1d we reveal the ability of anti-SHV FLABs to detect periplasmic SHV β-lactamases in a clinical *K. pneumoniae *isolate expressing the SHV-5 β-lactamase. Figure 1e and 1f demonstrate the visualization of SHV-1 β-lactamase in a laboratory strain of *E. coli *encoding and expressing SHV-1. In both instances, the FLABs readily detected the SHV β-lactamases. It is also noteworthy that this imaging technique reveals the morphology of the isolates with great definition.

Although PCR amplification remains the "gold standard" for the identification of *bla*_SHV _and other *bla *genes, FLABs may prove to be a rapid and easy "bench top" method. Our technique could be developed and used to rapidly test clinically important samples (e.g., blood and sputum) in a microbiology laboratory where the use of molecular methods (e.g., PCR/sequencing) is less feasible. By extension, interest will also be keen to assess the presence, distribution and regulation of β-lactamase expression in biofilms in device-associated infections.

When employing the FLABs method for β-lactamase detection, three important caveats should be kept in mind. Firstly, FLABs cannot distinguish between narrow-spectrum (e.g., SHV-1), broad-spectrum (e.g., SHV-11), and ESBLs (e.g., SHV-5 and SHV-12). Nevertheless, for Gram-negative organisms that do not express chromosomal SHV-type β-lactamases (e.g., *E. coli*, *Proteus *spp., *Enterobacter *spp.), evidence of SHV-type production is often associated with ESBLs. In this case, rapid identification of SHV enzymes could temper the use of cephalosporins and suggest an alternative antibiotic (e.g., carbapenems) in the critically ill patient with a serious infection. Secondly, low level β-lactamase expression due to either promoter mutations or gene copy number may affect the ability of FLABs to detect these enzymes. However, it has been shown that the limit of detection/sensitivity in ELISA experiments is at pg levels [13]. Thirdly, FLABs may cross react and detect the homologous LEN-type enzyme (possessed by some *K. pneumoniae*). In this study we were not able to rule out the possibility of cross-reaction between our FLABs and the LEN-type enzymes because we do not possess a highly-purified LEN-type β-lactamase and/or an isolate producing the *bla*_LEN _gene alone. Based on a comparison of amino acids sequences of SHV-1 and LEN-1 enzymes a homology of 90% was observed. We compared the immunogenic epitopes of SHV-1 to the amino acid sequence of LEN-1 [14]: the most higly recognized epitope showed 100% identity with the amino acid sequence of SHV-1 (data not shown). Therefore, it is possible that the LEN-type β-lactamase could be detected by our FLABs.

## Conclusion

We developed a rapid and accurate method of visualizing the SHV family of enzymes in clinical samples containing Gram-negative bacilli using fluorescein-labeled polyclonal antibodies. It has not escaped our attention that this approach can also be applied to other β-lactamase types and for different Gram-negative species. The application of this methodology for clinical samples could help to rapidly identify SHV production and promptly implement a more appropriate antibiotic therapy improving clinical outcome (e.g., length of hospital stay and mortality) of patients with serious infections due to different Gram-negative bacilli. The development of specific monoclonal antibodies would ensure more widespread application and supply. Further studies are planned to determine the ability of this method to detect SHV β-lactamase in a wide range of clinical isolates and to assess the localization of β-lactamases within the cell [17].

## Abbreviations

BSA: bovine serum albumin; ELISA: enzyme-linked immunosorbent assay; FISH: fluorescence *in situ *hybridization; FLAB: fluorescein-labeled polyclonal antibody; PBS: phosphate buffered saline; PCR: polymerase chain reaction; SHV: sulfhydryl reagent variable [18]; TEM: β-lactamase named after the patient Temoneira providing the first sample [18]; ESBL: extended-spectrum β-lactamase.

## Authors' contributions

AMH, KSK, NJD, and CRB involved in study design and execution of experiments. AMH, AE, and RAB study design and manuscript preparation. All authors read and approved the final manuscript.
